# Protocol for a Pilot Randomized Controlled Mixed Methods Feasibility Trial of a Culturally Adapted Peer Support and Self-Management Intervention for African Americans

**DOI:** 10.3390/pharmacy11010002

**Published:** 2022-12-21

**Authors:** Olayinka O. Shiyanbola, Martha Maurer, Meng-Jung Wen

**Affiliations:** 1 Division of Social and Administrative Sciences, School of Pharmacy, University of Wisconsin-Madison, Madison, WI 53705, USA; 2 Sonderegger Research Center for Improved Medication Outcomes, School of Pharmacy, University of Wisconsin-Madison, Madison, WI 53705, USA

**Keywords:** diabetes, self-management, peer support, medication adherence, mixed methods

## Abstract

Background: Due to diabetes disparities commonly seen among African Americans, it is important to address psychosocial and sociocultural barriers to medication adherence among African Americans with diabetes. Building on our prior work testing a culturally adapted peer supported diabetes self-management intervention for African Americans, this study will conduct a pilot randomized controlled feasibility trial that compares the culturally adapted intervention with a standard diabetes self-management program. Methods: Using an intervention mixed-methods design, the six-month trial will be conducted at two sites. Twenty-four African Americans with uncontrolled type 2 diabetes will be randomized to the intervention or control arm. Feasibility and acceptability outcomes in four domains (recruitment, intervention acceptability, intervention adherence, retention) will be collected. Primary clinical outcome (A1C), secondary outcome (medication adherence) and patient-specific psychosocial measures will be collected at baseline, 2 months, and 6 months. Document review, interview and focus groups will be used to gather qualitative data on feasibility and acceptability. Results: Expected results are that the trial protocol will be feasible to implement and acceptable for participants, and there will be a signal of clinically meaningful reduction in A1C and improvements in medication adherence. Conclusions: The results of this trial will inform a future powered large-scale randomized controlled trial testing the effectiveness of the culturally tailored intervention.

## 1. Introduction

African Americans are more likely to be diagnosed with type 2 diabetes, three times more likely to have diabetes complications, and twice as likely to die from diabetes, compared to all other racial/ethnic groups in the United States [1,2,3]. *Medication nonadherence* (defined as not taking medications as prescribed) is one primary reason for these health disparities. African Americans have poorer diabetes medication adherence compared to non-Hispanic whites, contributing to stroke, renal failure & lower-limb amputation [4,5]. Our research suggests that factors underlying these medication adherence disparities could be diabetes and medicine misbeliefs [6], and low self-efficacy, activation and engagement during patient-provider interactions influenced by discrimination experiences and provider distrust [7,8,9]. Though an elevated hemoglobin A1c (A1C) is driven by varying factors, studies document that medication adherence is one of the strongest predictors [10,11,12]. Hence, improving diabetes medication adherence leads to a reduction in A1C [8,11,13,14], and can reduce diabetes disparities commonly experienced by African Americans. There is a critical need for diabetes self-management interventions that address medication nonadherence and other self-management objectives, to reduce morbidity and mortality in this population [8,9,15,16,17].

A widely disseminated community-based program endorsed by the American Diabetes Association and the Center for Disease Prevention and Control is the evidence-based Disease Self-Management Education Program. This 6-week program, called Healthy Living with Diabetes (HLWD) in the state of Wisconsin, USA, addresses self-management but includes only brief content about medicine use [18,19,20,21]. HLWD is designed for all racial/ethnic groups with diabetes and does improve health outcomes. However, due to lack of culturally tailored content, and perhaps by not addressing medication adherence and diabetes and medication beliefs that may be culturally influenced, African Americans are underrepresented in their participation.

To address psychosocial and sociocultural barriers to medication adherence among African Americans with type 2 diabetes, we conducted a pilot trial to examine the acceptability and feasibility of a novel culturally tailored diabetes self-management intervention which focused on addressing these important barriers to medication adherence for African Americans—Peers’ Experiences in Communicating and Engaging in Healthy Living (Peers EXCEL) [22,23]. Peers EXCEL is based on our prior work where we explored African Americans’ reasons for diabetes medication nonadherence using literature review and focus groups. We found that reasons for nonadherence included medication beliefs such as concerns about medication side effects, doubts regarding whether medicines were safe and effective, and diabetes beliefs, including a denial of diabetes diagnosis, and misperception of the cause of diabetes [6,24]. Our study results showed that the perception of diabetes was influenced by African Americans’ sociocultural contexts including experiences of racial discrimination by providers [25]. Peer education is known to be effective in addressing negative beliefs among racial/ethnic minority groups and health beliefs [26,27]. Peer education from someone who is successfully managing their diabetes may enhance African Americans’ engagement in diabetes self-management. Several prior culturally adapted diabetes self-management programs for African Americans have conducted community and church-based programs [28], and focused on providing diabetes management education based on cultural appropriateness including using storytelling [5], focusing on African Americans’ diet and food preferences, and traditional values [29], and having the program be led by trusted community members [30]. Peers EXCEL integrates culturally tailored content on the important barriers to medication taking for African Americans, into the evidence-based HLWD program to improve the impact of diabetes self-management programs for African Americans [22,23].

Peers EXCEL provides an adapted diabetes self-management program for African Americans which includes group education sessions and one on one peer support. Group education includes the addition of new sessions that address misperceptions about diabetes and medicines influenced by racial discrimination/mistrust of providers, building self-efficacy, and provider communication. The additional one-on-one peer support occurs through race-congruent phone follow-up from other African Americans called Ambassadors. *Ambassadors* are African Americans with diabetes who are adherent to their medicines paired with African Americans with diabetes who are nonadherent (*Buddies*). Ambassadors’ interactions with their buddies focuses on addressing medicine and diabetes misperceptions, sharing experiences managing diabetes and medicines, and supporting self-efficacy and self-advocacy with their healthcare providers [31]. In the pilot trial of Peers EXCEL that examined feasibility and acceptability, we found the rates of recruitment were 80% for buddies and 100% for ambassadors. The rate at which the primary outcomes, medication adherence and A1C were retained was 75%. Participants mean completion rate was 13.4/17 of group sessions and phone calls with their ambassadors. About 84% of intervention phone calls were completed between the ambassadors and their buddies. We found no statistically significant differences in mean A1C and medication adherence. However, we found a clinically meaningful reduction (−0.7) in mean A1C at the inference point, 6-month follow up compared to the baseline. Based on the qualitative themes, participants reported the program to be culturally appropriate and acceptable with a perception of improved communication with their provider. They also reported that they learned strategies to assist with goal setting, and developed the confidence and motivation they needed for self-management [23].

Given these results, our next step is to conduct a pilot randomized controlled trial (RCT) of Peers EXCEL compared to HLWD to examine the feasibility and acceptability of the intervention and protocol and explore the benefits of the intervention in African Americans with uncontrolled diabetes using mixed methods.

## 2. Materials and Methods

### 2.1. Study Objectives and Design

**Figure 1:**
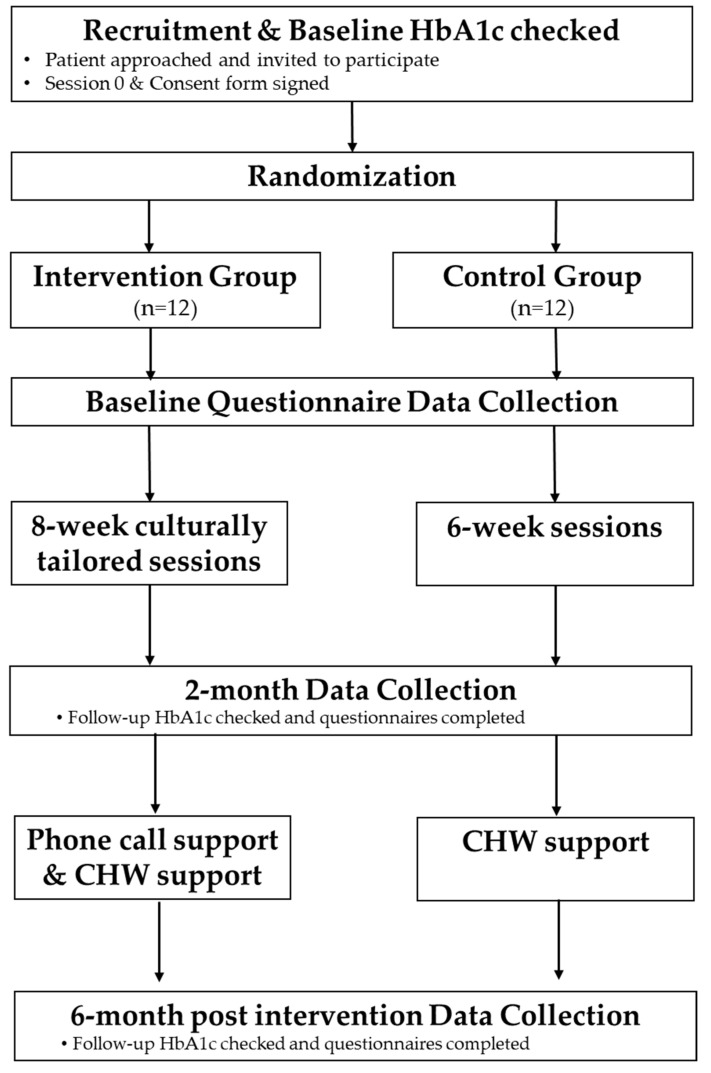
The Randomization Process.

The study objective is to evaluate the feasibility and acceptability of the trial protocol and the exploratory benefit of Peers EXCEL compared to HLWD using a pilot randomized controlled intervention mixed methods trial, among African Americans with uncontrolled diabetes. We will randomize participants to receive either Peers EXCEL or HLWD only. All individuals who agree to participate will be assigned a number and allocated to either the Peers EXCEL or HLWD group using computer-generated random numbers. The randomization process is illustrated in Figure 1. The study duration will be December 2022 until September 2023.

Our study aims are to:

(1) Evaluate if the intervention and protocol are feasible and acceptable. We will investigate if Peers EXCEL would be feasible to implement and be acceptable to African Americans with uncontrolled type 2 diabetes. Qualitative and quantitative data from multiple sources will be integrated to allow for meta-inferences about the feasibility of conducting a future large-scale effectiveness RCT.
(2) Pilot test Peers EXCEL to examine its effect in improving A1C and medication adherence. We hypothesize a signal of change in mean hemoglobin A1c that is clinically meaningful (≥0.6 reduction) for participants randomized to Peers EXCEL compared to participants randomized to HLWD at baseline, 2 months, and 6 months. We expect to see an improvement in medication adherence, assessed via self-report in the Peers EXCEL participants compared to the HLWD participants at 6 months.

We will use an intervention mixed methods design which will allow us to integrate complete and corroborated results from qualitative data before, during, and after the primary quantitative intervention trial [32,33]. In the recruitment and randomization phases, we will collect potential participants’ perceptions about participation in a trial. We will then integrate data from both qualitative and quantitative data sequentially at multiple points of the trial to examine the data collection, feasibility, acceptability, adherence, and retention of the intervention. Finally, participants’ experiences gathered from the follow-up interviews will allow us to further explain why and how the intervention practices and processes support their diabetes self-management and incorporate the skills into daily life. As well, qualitative findings will enhance the intervention trial to help refine the structures and processes for future interventions. We will strategically collect both qualitative and quantitative forms of data, which will allow for merging of the databases.

### 2.2. Theoretical Framework

The self-regulatory model (also called the common sense model of self-regulation) guided our preliminary work with African Americans with diabetes and established the foundation of our intervention, Peers EXCEL. This model theoretically explains the variation in patient responses to a chronic illness, including medication adherence [34,35,36]. According to the self-regulatory model, medication nonadherence occurs when there is no alignment between patients’ illness beliefs and medication recommendations (i.e., the prescribed medicine does not line up with the patient’s belief about the disease and the prescribed treatment they perceived is needed to control the disease) [37,38,39]. Taking diabetes medicines can be influenced by the patient beliefs about diabetes and the diabetes medicine.

Though the model focuses on modifiable constructs related to medication adherence and addresses illness and medication beliefs by improving these modifiable factors, it occurs via the mechanisms of the information-motivation-behavioral model [40]. This comprises providing information, which is an initial impetus to initiate the health behavior (medication adherence), motivation, which focuses on having a positive attitude towards medication adherence [41,42] and peer support to enhance engagement in medication-taking, and increasing behavioral skills towards self-efficacy and activation [41].

### 2.3. Study Setting

This pilot randomized controlled trial will be conducted in Madison, Wisconsin, USA at two community sites. The clinical trial is registered at: https://clinicaltrials.gov/ct2/show/NCT05527847 (accessed on 6 November 2022).

### 2.4. Participants

There are four groups of individuals involved in the pilot trial, but data analyses, and intervention outcomes will be collected principally from the HLWD and Peers EXCEL participants, i.e.,:

HLWD participants (control arm) (n = 12) –those who receive the HLWD content—who are African Americans with uncontrolled type 2 diabetes and nonadherent to their medicines.

Peers EXCEL participants (intervention arm) (n = 12)—those who receive the Peers EXCEL content—who are African Americans with uncontrolled type 2 diabetes and nonadherent to their medicines.

HLWD and Peers EXCEL participants’ inclusion criteria include: (1) Adults who are aged between 18 and 90 years old who are African American/Black with type 2 diabetes, and can speak and/or read English, (2) Self-report they have been prescribed at least one oral or injectable diabetes medicine, (3) Will be located in in the area geographically during the study period, (4) Self-reported medication nonadherence on the DOSE Nonadherence scale and (5) Most recent A1c value is ≥7.5% based on information collected by the research team at point of care A1C testing.

Exclusion criteria include: (1) If currently participating in a diabetes management or program focused on medication adherence, (2) Self-reported, schizophrenia, dementia, and untreated bipolar disorder or active substance use disorder (not having been in recovery for three or more months) (3) Older adults who report experiencing severe hypoglycemia in the past, and required medical assistance or the administration of glucagon

We will elicit feedback from individuals serving as Ambassadors and HLWD facilitators:

Peers EXCEL ambassadors (n = 4)—that is, those who relay intervention content via phone—who are African Americans with controlled diabetes (A1C ≤ 7.5%) and are adherent to their medication. Other inclusion criteria include (1) Adults who are aged between 18 and 90 years old who are African American/Black with type 2 diabetes, and can speak and/or read English, (2) Self-report they have been prescribed at least one oral or injectable diabetes medicine, (3) Being diagnosed with type 2 diabetes for at least one year (4) Will be located in the area geographically during the time of the study, (5) Being able to support another peer and track the phone conversations, and (6) Being prepared to attend all meetings and sessions focused on training them for their roles

HLWD facilitators must be African American/Black and either have diabetes themselves or have a close relationship with someone who does.

### 2.5. Procedures

#### 2.5.1. Participant Identification and Recruitment

Recruitment for all aims will be done in collaboration with two project community partners whose locations will also serve as the study community site for this proposal’s study’s implementation: (1) A nonprofit community-based organization serving older adults to provide a bridge to successful aging. This organization provides a variety of services and activities for older adults, and (2) A local church with a primarily African American/Black congregation.

For this project, the community partners will assist with informing potential ambassadors and participants about the study by distributing study flyers or via word of mouth, community presentations to their clients/congregation and referring these individuals to the study team for eligibility screening. They will also provide space and logistical support for holding the group education sessions for either the HLWD or Peers EXCEL program.

Through our established partnership with other community partners, we have been connected to churches, senior centers, barbershops, apartment complexes and community centers within African American communities. Hence, we will use word of mouth, flyer distribution, and program assistants actively meeting with potential clients in these organizations. Other recruitment approaches will include newspaper advertisements and social media postings.

#### 2.5.2. Screening

- Participants Screening: We will implement successful strategies used in our preliminary work and prior studies [22,23,43]. Eligible participants will complete a two-step screening process: (1) preliminary phone screening—A program assistant will ask if the individual meets the eligibility criteria including having a recent A1C value that showed ≥7.5%, and then (2) point-of-care A1C test to confirm that their A1C is ≥7.5%.
- Ambassador Screening for the Peers EXCEL arm: Based on our prior successful pilot study [22,23,43], after a ambassador candidate is known, a program assistant will complete a brief preliminary ambassador candidate screening form, ask the individual if they have had recent A1C values that are ≤7.5% and then, a point of care test to evaluate their A1C will be scheduled for verification. After these screenings are completed, the PI, program assistant, and research team members will meet with the candidate to explore other important characteristics, including their communication skills, and mentoring experiences. These characteristics will help inform the research team in the matching of an ambassador to a participant.

#### 2.5.3. The Control Arm (HLWD)

Participants will receive the standard widely disseminated diabetes self-management education classes for 6 weeks followed by community health worker (CHW) offer of support to receive resources related to social determinant of health barriers (Table 1).

Week 0—Baseline enrollment including a brief orientation about the study procedures, informed consent, and baseline data collection of surveys and A1C data.

Weeks 1–6 will consist of 6 separate group sessions. Diabetes self-management topics will be covered by 2 African American HLWD facilitators. Participants will meet one-time weekly for a 2½-h session, in a community setting such as community centers, senior center, or church.

Weeks 7–24: A CHW will offer each participant the option of care coordination and, if accepted, will help with social determinants of health barriers (e.g., transportation, food insecurity, housing, and employment issues; smoking cessation;) but does not cover the topic of medication adherence.

#### 2.5.4. The Intervention Arm (Peers EXCEL)

In addition to the 6-week group education classes on diabetes self-management, (HLWD), participants will receive race-congruent peer support and culturally tailored intervention content addressing sociocultural barriers to medication nonadherence among African Americans (see Table 1). This additional content will include: (1) two group education sessions discussing diabetes and medication beliefs, racial/discrimination/mistrust, and how to build positive relationships with providers for enhanced communication, and (2) phone support from ambassadors occurring every week for weeks 3–8, then bi-weekly for weeks 9–12.

Week 0—Baseline enrollment including a brief orientation about the study procedures, informed consent, and baseline data collection of surveys and A1C data.

Weeks 1–8 will consist of 8 group sessions, including one led by a pharmacist- and one session led by a healthcare provider to discuss diabetes and medication beliefs, and provider distrust and improved communication. Community engagement and stakeholder feedback from our prior studies emphasized the importance of a provider-led session to help develop trust in the community and enhance equity [44,45]. Topics focused on diabetes self-management from HLWD will be covered by 2 African American/Black HLWD-trained facilitators. As well, the ambassadors will attend each group session together with their assigned peer participant, during which they will interact with the individual, allowing them to learn together and build social interactions. Additionally, ambassadors will complete phone follow-ups every week with their peer to further discuss the group education sessions and phone intervention topics.

Weeks 9–16: In addition to the CHWs’ initial offers of assistance with social determinant of health barriers, ambassadors will conduct bi-weekly ‘check-in’ calls with their participant to further support the maintenance of their goals. As well, they will review the set goals and the progress made together, discuss any barriers the individual is having regarding meeting the goals, and collectively problem-solve ways of addressing the barriers. Peer participants will be able to call their ambassadors for support during these months, if needed, and the ambassadors will take notes about the phone call content.

Weeks 17–24: Ambassadors will make calls to peer participants monthly to emphasize content they had learnt and discussed during the group sessions and provide assistance with maintaining goals or resetting them, if fitting.

##### Training of Ambassadors

Before the implementation of Peers EXCEL, ambassadors will be prepared for their role as they will attend a 3-day training program (9-h) facilitated by the research team with stakeholder engagement experience for >5 years. The PI and team have trained and facilitated several lay advisory board meetings [44,45]. The first meeting will be an orientation. Subsequent meetings will prepare ambassadors for the implementation of specific components of Peers EXCEL, including the process for the phone calls. Ambassadors will be guided on how to document their phone conversations, phone call attempts and completed calls and record the date, approximate duration of contact, and intervention content topics discussed. Role plays will be used during the training. These training meetings will be guided by a manual and toolkit prepared from our prior work focusing on supportive non-judgmental communication and peer support, active and reflective listening, and goal setting [22,23,43,44,45]. Similar to our prior work, ambassadors will complete a community-based training on research ethics, prior to their training for Peers EXCEL. To ensure the standardization of the training provided, 100% attendance of all training sessions and active participation during the training will be required. Individuals who do not complete the training will not be engaged in the role of an ambassador and will be excluded from participating.

### 2.6. Data Collection

Quantitative data will be collected using in-person surveys at baseline, 2 months, and 6 months. Qualitative interviews and focus groups will be conducted with all study participants, HLWD facilitators, and the ambassadors to understand their experiences with the intervention and their feedback on the program. These interviews will be conducted at 2 and 6 months. Field notes and documents from program assistants will be reviewed for data abstraction.

- Surveys: A ~25 min longitudinal survey will be administered to measure self-reported medication adherence (secondary outcome) and patient-reported psychosocial factors to all participants at baseline, 2 and 6 months. The survey will be administered to each person in-person and orally during the data collection time periods, to account for people having low literacy or cognitive impairment. Surveys including reliable and validated survey questionnaires will be given to participants to assess beliefs about diabetes, self-efficacy, patient activation, and perceived quality of patient-provider communication and A1C tests at baseline, 2 months, and 6 months assessing the feasibility of gathering outcome data.
- Qualitative interview: In-person semi-structured ~25 min interviews will be conducted with all participants immediately after completing either the HLWD or Peers EXCEL group sessions and again at the end of the 6-month intervention to explore their feedback on the programs, the potential impact on changes in medication adherence and other outcomes. Participants’ inclusion and exclusion criteria will be similar to the trial. The qualitative interviews will be on-going until we reach data saturation. Sample interview questions are listed in Table 2.
- Focus groups: All ambassadors will be asked to participate in a focus group lasting 90 min which will be completed at the end the 8-week Peers EXCEL group sessions and again at the end of the intervention. Focus groups allow for a range of responses from participants compared to one-on-one interviews and ambassadors can generate new ideas and feedback for each other, which may not occur in an interview. Questions will focus on feedback about the feasibility outcomes: experiences with the process we used for recruitment, trainings they received, sustaining their participation during the Peers EXCEL intervention, and ideas for how to make the work of an ambassador easier and manageable. Sample focus group topic guide questions are listed in Table 3.

**Table 2:** Sample Questions for Participants.

Overall program experience/benefit Tell me about your experience so far with the program you participated in. Tell me your thoughts about the cultural appropriateness of the program? PROBE: For example, how were the suggestions about diet relevant for you as an African American? PROBE: How did the discussion of managing stress relate to what’s going on in your life or the lives of African American/Blacks? PROBE: How did the program allow for opportunities to discuss topics or examples relevant for African Americans/Blacks? PROBE: How were the book and session activities relevant for you or African American/Blacks? Thinking ahead to the future, would you be willing to attend the group sessions again? If not, could you let us know why?
Feedback about healthcare professional group education sessions (Intervention group only) What did you learn from the doctor’s/pharmacist’s session? What did you put into practice based on what you learned in that session?
Feedback about the diabetes self-management topic sessions What was the most useful information you learned from the sessions on managing diabetes? What are your thoughts about the way the sessions on managing diabetes were delivered? How do you think that part of the program could be improved?
Feedback about the interactions with Ambassadors (Intervention group only) Tell us about what you discussed with your ambassador during the first two phone calls. PROBE: What went well with the phone calls? PROBE: What were some of the challenges? PROBE: How could they be improved? How was it helpful to talk with your ambassador? What kind of support did you receive? What tips or resources were most useful? If participant response is that it wasn’t helpful, then ask: c. How was talking with your ambassador not helpful for you?

**Table 3:** Sample focus group guide for Peers EXCEL Ambassadors.

Overall program experience Tell me about your experience so far with Peers EXCEL. In what ways did the program work? In what ways did the program not work? Did it create new challenges? What was beneficial about serving as an ambassador for Peers EXCEL? What were the hardest parts about being an ambassador for Peers EXCEL?
Feedback about the HLWD sessions Tell us your thoughts about the information on managing diabetes that was provided as part of the program. What are your thoughts about the delivery of the diabetes management sessions by the two facilitators? How do you think that the diabetes management sessions could be improved?
Feedback about phone calls with Peers EXCEL participants Tell us about what you discussed with your paired participant during the phone calls after the diabetes management sessions ended. Tell us about your experience with the phone calls with your participant. What are your thoughts about the last two calls with your participant that were once a month?
Feedback about further training and support from the research team What could be done to improve the support the research team provided to you as an ambassador during the period only phone calls were made? Are there things that the research team didn’t think about that would be important to address during these months when only phone calls made? What could the study team do to better support ambassadors throughout the program? What could the study team do to better train and prepare ambassadors for their role in the program?

#### 2.6.1. Measures

All study outcome measures, collected at baseline, 2 and 6 months, are listed in Table 4.

The primary clinical study outcome, A1C, will be measured using A1cNow+, the National Glycohemoglobin Standardization Program Certified, which is a CLIA-waived system that uses a finger stick test. The American Diabetes Association considers this clinical measure an indication of success for diabetes self-management. A1C assessments will be assessed by a clinical staff. They will occur in private community locations at 2 sites.

The secondary study outcome, medication adherence, will be assessed using a self-reported medication adherence scale, the 3-item Extent of Nonadherence domain in the reliable and validated DOSE-Nonadherence survey. This measure screens for nonadherence and is calculated by computing the mean of the 3 items (score range is 1–5). Participants with mean scores of 3 (i.e., scoring “1” on each of the 3 items) will be classified as nonadherent, while participants with mean scores >3 will be classified as adherent [46,47].

Other outcomes: Several validated measures will provide data on the effect of proposed theoretical constructs on Peers EXCEL and its impact on the patient-specific psychosocial constructs. These measures are listed in Table 4.

Demographic/clinical factors include age, gender, self-reported health, depressive symptoms, self-reported cardiovascular event, hospitalization, emergency room visit due to diabetes complications, and number of prescription medications used.

Feasibility and acceptability outcomes: Outcomes to be evaluated will include recruiting of ambassadors, HLWD and Peers EXCEL participants, attrition, and participation in program components. We will record how many participants were screened to meet recruitment goals, document which recruitment goals strategies work best, and the length of time needed to recruit. We will also document the number of participants who attended each group sessions, and the number of participants who continued their participation in the intervention will be compared with the numbers we recruited at the beginning. For participants in the intervention arm, phone use will be tracked weekly by asking participants the amount of time they spent on the phone with their ambassador.

#### 2.6.2. Mixed Methods Integration

Qualitative and quantitative data from multiple sources will be integrated to allow for meta-inferences about the feasibility of conducting a future large-scale effectiveness randomized controlled trial. Sources of quantitative data will include study administrative records (to assess feasibility of recruitment, intervention adherence, and retention); surveys completed after each session (to assess acceptability of different features of the intervention, including the presenter/HLWD facilitator, content, structure, and group session logistics); and surveys completed at the post-intervention assessment (to assess acceptability and adherence to data collection procedures and overall intervention acceptability). Qualitative data on acceptability of data collection will also be obtained by the open-ended questions in the post-intervention semi-structured interviews and document review. To obtain qualitative data on the other four feasibility domains of greatest interest—recruitment, intervention acceptability, intervention adherence, and retention—we will hold semi-structured interviews within two weeks following the final HLWD group session (scheduled to take place after all participant assessments) and at the end of the 6-month intervention. We will also evaluate documents and field notes data from study team calls with participants (to explore reasons for declining participation related to recruitment and retention). We will employ various strategies for integrating quantitative and qualitative outcomes. Qualitative data may provide insight into reasons why corresponding quantitative metrics are lagging or provide convergent and contextualized evidence in support of positive quantitative metrics.

#### 2.6.3. Intervention Fidelity

Evaluating content fidelity involves determining whether content will be delivered as intended. Study team members will use a 14-item program monitoring tool to assess the HLWD facilitator content fidelity in 5 areas: Environment (e.g., room and seating for the group session), Content (e.g., time used for the presentation and clarity of content), Presenter, Program Delivery, and Methods/Materials (e.g., educational strategies used). An external evaluator will address things such as the facilitators’ adherence to the program guide, how they assisted in participants’ problem-solving and brainstorming activities, assigning of homework, and helping participants develop action plans to promote self-efficacy [54,55]. Peers EXCEL group sessions fidelity will focus on delivery of culturally tailored content.

For the Peers EXCEL arm, we will also monitor fidelity similar to the process used in prior studies [22,23,43]: (1) group education sessions with participants will be audio-recorded to examine intervention implementation, (2) phone calls every week from program assistants to ambassadors to discuss content and rate of phone calls with peer participants, and (3) bi-weekly calls from program assistants to document how the peer phone calls are going. All group sessions will be audio recorded to avoid a Hawthorne effect, where knowing they are being recorded for certain sessions enhances the facilitators’ actions during that session. We will then listen to a randomly chosen set of sessions. The PI, study coordinator, program assistant, and the facilitators will assess for fidelity by discussing the sessions and creating an opportunity to provide feedback on each session.

### 2.7. Data Analysis

- Quantitative. Paired *t*-tests (or a non-parametric corresponding test such as Wilcoxon rank sum test) will examine pre- vs. post-intervention changes in participant’s A1C, medication adherence, and other psychosocial outcomes across groups to examine a signal of change. We will use descriptive statistics to calculate ambassadors’ feasibility measures, HLWD and Peers EXCEL participants, including ambassador recruitment, ambassador attrition, and extent of ambassador participation in sessions related to their training and intervention. We will consider the recruitment approach as feasible if: there is recruitment of all ambassadors and participants as planned, attrition for both ambassadors and participants is less than or equal to 10%, and the rate for ambassador and participant participation is equal to or higher than 80%.
- Qualitative. Interviews and focus groups will be audio-recorded and transcribed. Research assistants will code transcripts inductively using NVivo v 12 and conduct qualitative content analysis [56]. Qualitative content analysis will be used to organize the themes. All transcribed transcripts will be read initially for data immersion, taking time to read all the data line by line. Then, the codes and themes will be developed and organized with a conceptualization of how the themes are all lined together in the data. We will compare all themes exploring if there are similarities, interconnections, and/or differences across all themes. We will continue all data analysis until we get to theoretical and there are no more new dimensions in the data [57,58,59]. We will establish rigor of the data and explore the trustworthiness of the data analysis process using Lincoln and Guba (1985) four general criteria [60]. These are credibility, transferability, dependability, and confirmability. For credibility, two research assistants will code the transcripts independently—investigator triangulation (i.e., multiple coders involved in the data analysis), discuss similarities and divergences, and reach agreement by consensus before the final data interpretation. We will member check with participants interested in being part of the process to confirm if our interpretation is salient/credible—to check for resonance with participant experiences. Confirmability, objectivity/potential congruence between researchers. To ensure our findings are based on our participants’ responses and not any personal motivations or personal bias from our research team, after coding, all similarities and divergences will be discussed. Agreement will be reached on codes before results interpretation. Transferability, the scope to which results are applicable to other contexts. We will purposively sample individuals with varied intervention experiences and use detailed descriptions to show how the research study’s findings may be applicable in other contexts, circumstances, and situations. Dependability—the ability to achieve consistent findings if the study is done as described. We will create and report a detailed audit trail of our process throughout the analysis process. Documents and field notes data will also be analyzed using content analysis.
- Mixed. After analyzing the quantitative and qualitative data separately, the mean score differences, statistical effect sizes, and themes will be compared in the context of the feasibility, acceptability, and primary and secondary outcomes. The results from both phases will be interpreted together in a joint display to aid a meta-inference of the merged results.

## 3. Expected Results

It is expected that the intervention trial protocol will be feasible to implement and be acceptable to African Americans with type 2 diabetes. Finally, we expect that participants who complete the Peers EXCEL intervention will have a signal of change in mean A1C that is clinically meaningful (≥0.6 reduction) compared to participants randomized to HLWD at baseline, 2 months, and 6 months. The primary outcome is to see a reduction in A1C, while the secondary outcome will be to determine if there is an improvement in medication adherence, assessed via self-report in the Peers EXCEL participants compared to the HLWD participants at 6 months.

### 3.1. Limitations

Despite this study’s potential strengths, there are some potential limitations and anticipated challenges. Our research team has planned for how to address these potential issues, when possible. For example, it is likely that peer matching will not work initially, possibly because of the unavailability of an ambassador. If this occurs, we will use our established protocol from our prior studies for reassignment. Though not experienced in our prior work, recruitment retention of ambassadors may be an issue. To minimize this possibility, we will initially match ambassadors and their peer participants on a 1:3 ratio and allow 1:4 if needed. Recruiting enough African Americans may be challenging. Therefore, the research team has developed strong partnerships with several community partners in the study location. Early in the process of designing the study, the research team met with the community partners to present the study idea and plan and received their commitment where they would assist with recruiting participants. Another potential issue we could face is retaining the study participants during the study period. To assist with increasing participant retention, the research team will have check-in phone calls with each participant once every week. We currently plan to have face-to-face group sessions for the intervention. If a virtual approach is needed, both the HLWD and Peers EXCEL programs have experience being held virtually.

### 3.2. Implications and Future Research

This pilot randomized controlled trial has the potential for further understanding if a culturally tailored intervention integrating evidence-based diabetes self-management content with race-congruent peer support improves hemoglobin A1c, medication adherence, and other psychosocial and behavioral barriers to medication adherence compared to a standard evidence-based diabetes self-management program. Findings from this study may address an unmet critical need to provide culturally tailored educational content and peer support to a patient population historically beset by harms related to uncontrolled diabetes. Building upon a widely used, evidence-based program, we anticipate that the intervention has the potential to be disseminated nationally in the United States to reduce diabetes mortality and morbidity in African Americans. Future research can expand on this study by testing the effectiveness of the culturally tailored intervention in a large powered randomized controlled trial.

## 4. Conclusions

This protocol paper described the design and proposed pilot randomized controlled trial to examine the acceptability, feasibility, and signal of benefit of a culturally adapted self-management intervention for African Americans with uncontrolled type 2 diabetes. To our knowledge, this is the first study to examine whether an intervention with amplified focus on medication adherence and culturally tailored race-congruent peer support combined with evidence-based diabetes self-management group education optimizes glycemic control and medication adherence among African Americans.

## Figures and Tables

**Table 1:** Intervention Content for Intervention Arm (Peers EXCEL) and Control Arm (HLWD).

Intervention Content	Weeks											
	1	2	3	4	5	6	7	8	9	14	19	24
Group sessions of beliefs about diabetes, provider mistrust and pharmacist communication	*	*										
Group sessions of diabetes self-management, healthy eating, problem solving, exercise, communication, medication, cultural experiences, discussing diabetes with family			*	*	*	*	*	*				
			#	#	#	#	#	#				
Referral to community health worker, if requested									*	*	*	*
									#	#	#	#
Peer-based phone call support									*	*	*	*

* Intervention Arm (Peers EXCEL Program); # Control Arm (Healthy Living with Diabetes Program).

**Table 4:** Measures for Study Outcomes.

Construct	Measure	Baseline	2 Months	6 Months
Primary Clinical Outcome				
Blood glucose	Hemoglobin A1c (A1C)	×	×	×
Secondary Study Outcome				
Medication adherence	DOSE-Nonadherence survey [46,47], extent of nonadherence domain	×	×	×
Other Measures				
Diabetes-health beliefs	Brief Illness Perception Questionnaire [48]	×	×	×
Beliefs about diabetes medicines	Beliefs about Medicines Questionnaire [35]	×	×	×
Diabetes and medication self-efficacy	Diabetes Empowerment Scale—Short Form [49] Self-Efficacy for Adherence to Medication Use Scale [50]	×	×	×
Diabetes distress	Diabetes Distress Scale (DDS-2) [51]			
Patient-provider communication	Patient’s Perceived Involvement in Care Scale [52]	×	×	×
Patient activation	Patient Activation Measure [53]	×	×	×

## Data Availability

Not applicable.

## References

[1] Getz L. (2009). African Americans and Diabetes—Educate to Eliminate Disparities Among Minorities. Today’s Dietitian. May.

[2] Perneger T.V., Brancati F.L., Whelton P.K., Klag M.J. (1994). End-stage renal disease attributable to diabetes mellitus. Ann. Intern. Med..

[3] U.S. Department of Health and Human Services Office of Minority Health (2021). Diabetes and African Americans. https://minorityhealth.hhs.gov/omh/browse.aspx?lvl=4&lvlid=18.

[4] Chow E.A., Foster H., Gonzalez V., McIver L. (2012). The Disparate Impact of Diabetes on Racial/Ethnic Minority Populations. Clin. Diabetes.

[5] Williams I.C., Utz S.W., Hinton I., Yan G., Jones R., Reid K. (2014). Enhancing diabetes self-care among rural African Americans with diabetes: Results of a two-year culturally tailored intervention. Diabetes Educ..

[6] Shiyanbola O.O., Brown C.M., Ward E.C. (2018). “I did not want to take that medicine”: African-Americans’ reasons for diabetes medication nonadherence and perceived solutions for enhancing adherence. Patient Prefer. Adherence.

[7] Mann D.M., Ponieman D., Leventhal H., Halm E.A. (2009). Predictors of adherence to diabetes medications: The role of disease and medication beliefs. J. Behav. Med..

[8] Schectman J.M., Nadkarni M.M., Voss J.D. (2002). The association between diabetes metabolic control and drug adherence in an indigent population. Diabetes Care.

[9] Shenolikar R.A., Balkrishnan R., Camacho F.T., Whitmire J.T., Anderson R.T. (2006). Race and medication adherence in Medicaid enrollees with type-2 diabetes. J. Natl. Med. Assoc..

[10] Ho P.M., Rumsfeld J.S., Masoudi F.A., McClure D.L., Plomondon M.E., Steiner J.F., Magid D.J. (2006). Effect of medication nonadherence on hospitalization and mortality among patients with diabetes mellitus. Arch. Intern. Med..

[11] Lawrence D.B., Ragucci K.R., Long L.B., Parris B.S., Helfer L.A. (2006). Relationship of oral antihyperglycemic (sulfonylurea or metformin) medication adherence and hemoglobin A1c goal attainment for HMO patients enrolled in a diabetes disease management program. J. Manag. Care Pharm. JMCP.

[12] Rozenfeld Y., Hunt J.S., Plauschinat C., Wong K.S. (2008). Oral antidiabetic medication adherence and glycemic control in managed care. Am. J. Manag. Care.

[13] Hill-Briggs F., Gary T.L., Bone L.R., Hill M.N., Levine D.M., Brancati F.L. (2005). Medication adherence and diabetes control in urban African Americans with type 2 diabetes. Health Psychol..

[14] Krapek K., King K., Warren S.S., George K.G., Caputo D.A., Mihelich K., Holst E.M., Nichol M.B., Shi S.G., Livengood K.B. (2004). Medication adherence and associated hemoglobin A1c in type 2 diabetes. Ann. Pharmacother..

[15] Heisler M. (2007). Overview of Peer Support Models to Improve Diabetes Self-Management and Clinical Outcomes. Diabetes Spectr..

[16] Hu D., Juarez D.T., Yeboah M., Castillo T.P. (2014). Interventions to increase medication adherence in African-American and Latino populations: A literature review. Hawai’i J. Med. Public Health.

[17] Patel I., Erickson S.R., Caldwell C.H., Woolford S.J., Bagozzi R.P., Chang J., Balkrishnan R. (2016). Predictors of medication adherence and persistence in Medicaid enrollees with developmental disabilities and type 2 diabetes. Res. Soc. Adm. Pharm. RSAP.

[18] Lorig K., Ritter P.L., Ory M.G., Whitelaw N. (2013). Effectiveness of a generic chronic disease self-management program for people with type 2 diabetes: A translation study. Diabetes Educ..

[19] Lorig K., Ritter P.L., Turner R.M., English K., Laurent D.D., Greenberg J. (2016). A Diabetes Self-Management Program: 12-Month Outcome Sustainability From a Nonreinforced Pragmatic Trial. J. Med. Internet Res..

[20] Lorig K., Ritter P.L., Villa F., Piette J.D. (2008). Spanish diabetes self-management with and without automated telephone reinforcement: Two randomized trials. Diabetes Care.

[21] Lorig K., Ritter P.L., Villa F.J., Armas J. (2009). Community-based peer-led diabetes self-management: A randomized trial. Diabetes Educ..

[22] Shiyanbola O.O., Tarfa A., Song A., Sharp L.K., Ward E. (2019). Preliminary Feasibility of a Peer-supported Diabetes Medication Adherence Intervention for African Americans. Health Behav. Policy Rev..

[23] Shiyanbola O.O., Maurer M., Schwerer L., Sarkarati N., Wen M.J., Salihu E.Y., Nordin J., Xiong P., Egbujor U.M., Williams S.D. (2022). A Culturally Tailored Diabetes Self-Management Intervention Incorporating Race-Congruent Peer Support to Address Beliefs, Medication Adherence and Diabetes Control in African Americans: A Pilot Feasibility Study. Patient Prefer. Adherence.

[24] Shiyanbola O.O., Ward E.C., Brown C.M. (2018). Utilizing the common sense model to explore African Americans’ perception of type 2 diabetes: A qualitative study. PLoS ONE.

[25] Shiyanbola O.O., Ward E., Brown C. (2018). Sociocultural Influences on African Americans’ Representations of Type 2 Diabetes: A Qualitative Study. Ethn. Dis..

[26] Peterson E.B., Chou W.S., Rising C., Gaysynsky A. (2020). The Role and Impact of Health Literacy on Peer-to-Peer Health Communication. Stud. Health Technol. Inform..

[27] Salud M.A., Gallardo J.I., Dineros J.A., Gammad A.F., Basilio J., Borja V., Iellamo A., Worobec L., Sobel H., Olivé J.M. (2009). People’s initiative to counteract misinformation and marketing practices: The Pembo, Philippines, breastfeeding experience, 2006. J. Hum. Lact..

[28] Collins-McNeil J., Edwards C.L., Batch B.C., Benbow D., McDougald C.S., Sharpe D. (2012). A culturally targeted self-management program for African Americans with type 2 diabetes mellitus. Can. J. Nurs. Res..

[29] Peña-Purcell N.C., Jiang L., Ory M.G., Hollingsworth R. (2015). Translating an evidence-based diabetes education approach into rural african-american communities: The “wisdom, power, control” program. Diabetes Spectr..

[30] Samuel-Hodge C.D., Keyserling T.C., France R., Ingram A.F., Johnston L.F., Pullen Davis L., Davis G., Cole A.S. (2006). A church-based diabetes self-management education program for African Americans with type 2 diabetes. Prev. Chronic Dis..

[31] Wen M.J., Maurer M., Schwerer L., Sarkarati N., Egbujor U.M., Nordin J., Williams S.D., Liu Y., Shiyanbola O.O. (2022). Perspectives on a Novel Culturally Tailored Diabetes Self-Management Program for African Americans: A Qualitative Study of Healthcare Professionals and Organizational Leaders. Int. J. Environ. Res. Public Health.

[32] Creswell J.W., Clark V.L.P. (2017). Designing and Conducting Mixed Methods Research.

[33] Rao D., Shiyanbola O.O. (2022). Best practices for conducting and writing mixed methods research in social pharmacy. Res. Soc. Adm. Pharm. RSAP.

[34] Byer B., Myers L.B. (2000). Psychological correlates of adherence to medication in asthma. Psychol Health Med..

[35] Horne R., Weinman J. (1999). Patients’ beliefs about prescribed medicines and their role in adherence to treatment in chronic physical illness. J. Psychosom. Res..

[36] Jörgensen T.M., Andersson K.A., Mårdby A.C. (2006). Beliefs about medicines among Swedish pharmacy employees. Pharm. World Sci. PWS.

[37] Brown C., Battista D.R., Bruehlman R., Sereika S.S., Thase M.E., Dunbar-Jacob J. (2005). Beliefs About Antidepressant Medications in Primary Care Patients: Relationship to Self-Reported Adherence. Med. Care.

[38] Horne R., Weinman J. (2002). Self-regulation and Self-management in Asthma: Exploring The Role of Illness Perceptions and Treatment Beliefs in Explaining Non-adherence to Preventer Medication. Psychol Health.

[39] Ross S., Walker A., MacLeod M.J. (2004). Patient compliance in hypertension: Role of illness perceptions and treatment beliefs. J. Hum. Hypertens..

[40] Misovich S.J., Martinez T., Fisher J.D., Bryan A., Catapano N. (2003). Predicting Breast Self-Examination: A Test of the Information-Motivation-Behavioral Skills Model1. J. Appl. Soc. Psychol..

[41] Fisher W.A., Fisher J.D., Harman J. (2003). The Information–Motivation–Behavioral skills model as a general model of health behavior change: Theoretical approaches to individual-level change. Social Psychological Foundations of Health.

[42] Osborn C.Y. (2006). Using the IMB Model of Health Behavior Change to Promote Self-Management Behaviors in Puerto Ricans with Diabetes.

[43] Shiyanbola O.O., Maurer M., Ward E.C., Sharp L., Lee J., Tarfa A. (2020). Protocol for partnering with peers intervention to improve medication adherence among African Americans with Type 2 Diabetes. medRxiv.

[44] Maurer M.A., Shiyanbola O.O., Mott M.L., Means J. (2022). Engaging Patient Advisory Boards of African American Community Members with Type 2 Diabetes in Implementing and Refining a Peer-Led Medication Adherence Intervention. Pharmacy.

[45] Shiyanbola O.O., Kaiser B.L., Thomas G.R., Tarfa A. (2021). Preliminary engagement of a patient advisory board of African American community members with type 2 diabetes in a peer-led medication adherence intervention. Res. Involv. Engagem..

[46] Cornelius T., Voils C.I., Umland R.C., Kronish I.M. (2019). Validity Of The Self-Reported Domains Of Subjective Extent Of Nonadherence (DOSE-Nonadherence) Scale In Comparison With Electronically Monitored Adherence To Cardiovascular Medications. Patient Prefer Adherence.

[47] Voils C.I., King H.A., Thorpe C.T., Blalock D.V., Kronish I.M., Reeve B.B., Boatright C., Gellad Z.F. (2019). Content Validity and Reliability of a Self-Report Measure of Medication Nonadherence in Hepatitis C Treatment. Dig. Dis. Sci..

[48] Broadbent E., Petrie K.J., Main J., Weinman J. (2006). The brief illness perception questionnaire. J. Psychosom. Res..

[49] Anderson R.M., Funnell M.M., Fitzgerald J.T., Marrero D.G. (2000). The Diabetes Empowerment Scale: A measure of psychosocial self-efficacy. Diabetes Care.

[50] Risser J., Jacobson T.A., Kripalani S. (2007). Development and psychometric evaluation of the Self-efficacy for Appropriate Medication Use Scale (SEAMS) in low-literacy patients with chronic disease. J. Nurs. Meas..

[51] Fisher L., Glasgow R.E., Mullan J.T., Skaff M.M., Polonsky W.H. (2008). Development of a brief diabetes distress screening instrument. Ann. Fam. Med..

[52] Lerman C.E., Brody D.S., Caputo G.C., Smith D.G., Lazaro C.G., Wolfson H.G. (1990). Patients’ Perceived Involvement in Care Scale: Relationship to attitudes about illness and medical care. J. Gen. Intern. Med..

[53] Hibbard J.H., Stockard J., Mahoney E.R., Tusler M. (2004). Development of the Patient Activation Measure (PAM): Conceptualizing and measuring activation in patients and consumers. Health Serv. Res..

[54] Health Innovation Program Improving Diabetes Self-Management. https://hip.wisc.edu/hlwd.

[55] Wisconsin Institute for Health Aging Healthy Living with Diabetes. https://wihealthyaging.org/programs/live-well-programs/hlwd/.

[56] Hsieh H.F., Shannon S.E. (2005). Three approaches to qualitative content analysis. Qual. Health Res..

[57] Charmaz K., Belgrave L.L. (2012). The SAGE Handbook of Interview Research: The Complexity of the Craft.

[58] Pope C., Ziebland S., Mays N. (2000). Qualitative research in health care. Analysing qualitative data. BMJ (Clin. Res. Ed.).

[59] Richards L. (2020). Handling Qualitative Data: A Practical Guide.

[60] Lincoln Y.S., Guba Y.S.L.E.G., Guba E.G., Publishing S. (1985). Naturalistic Inquiry.

